# CASCADE: criticality avalanche spike cross-platform analysis detection engine, a multi-manufacturer MEA bash analysis pipeline

**DOI:** 10.1093/bioinformatics/btag570

**Published:** 2026-07-29

**Authors:** Forbes Avila, Jin Kim, Jong-Chan Park, Rian Kang, Hyunsu Lee, Sun-Hyun Park

**Affiliations:** Center for Alternatives to Animal Testing, Korea Institute of Toxicology, Yuseong, Daejeon 34114, Republic of Korea; Human and Environmental Toxicology, University of Science and Technology (UST), Yuseong, Daejeon 34113, Republic of Korea; Department of Physiology, College of Medicine, Soonchunhyang University, Cheonan 31151, Republic of Korea; Institute for Molecular Metabolism Innovation, Soonchunhyang University, Asan 31538, Republic of Korea; Department of Biophysics, Institute of Quantum Biophysics, Sungkyunkwan University, Suwon 16419, Republic of Korea; Department of MetaBioHealth, Sungkyunkwan University, Suwon 16419, Republic of Korea; Department of Biopharmaceutical Convergence, Sungkyunkwan University, Suwon 16419, Republic of Korea; Department of Biopharmaceutical Convergence, Sungkyunkwan University, Suwon 16419, Republic of Korea; Department of Physiology, School of Medicine, Pusan National University, Yangsan-si 50612, Republic of Korea; Research Institute for Convergence of Biomedical Science and Technology, Pusan National University Yangsan Hospital, Yangsan-si 50612, Republic of Korea; Medical Research Institute, School of Medicine, Pusan National University, Yangsan-si 50612, Republic of Korea; Center for Alternatives to Animal Testing, Korea Institute of Toxicology, Yuseong, Daejeon 34114, Republic of Korea; Human and Environmental Toxicology, University of Science and Technology (UST), Yuseong, Daejeon 34113, Republic of Korea

## Abstract

**Summary:**

Multi-electrode array (MEA) recordings are widely utilized to characterize neuronal network activity. However, currently each manufacturer distributes proprietary software that operates exclusively on its own file format. Since no cross-platform, open-source pipeline currently exists. Thus, we created CASCADE (Criticality Avalanche Spike Cross-platform Analysis Detection Engine), a Python-based command-line (bash) pipeline to analyze MEA recordings from 3Brain, Axion Biosystems, Cortical Labs, Maxwell Biosystems, and Multi-Channel Systems MEA devices. CASCADE measures the standard parameters such as: network, burst, firing rate frequency, and also measures criticality and avalanches. CASCADE criticality calculation was benchmarked across six MEA recordings from five manufacturers, resulting in comparable values because all recordings were analyzed using identical computational methods regardless of manufacturer file format. Thus, CASCADE provides a flexible and robust MEA analysis pipeline that can also analyze neuronal avalanches and criticality from multiple MEA devices from different manufacturers.

**Availability and implementation:**

CASCADE is freely available at GitHub repository (https://github.com/FA387/cascade-mea) and can be installed via PyPI (pip install cascade-mea). The source code is archived at Zenodo (https://doi.org/10.5281/zenodo.20840795). Data used in this work is available at Zenodo repository (https://doi.org/10.5281/zenodo.19161615). The data underlying this article are available in the article and in its online supplementary material.

## Graphical Abstract

**Figure:**
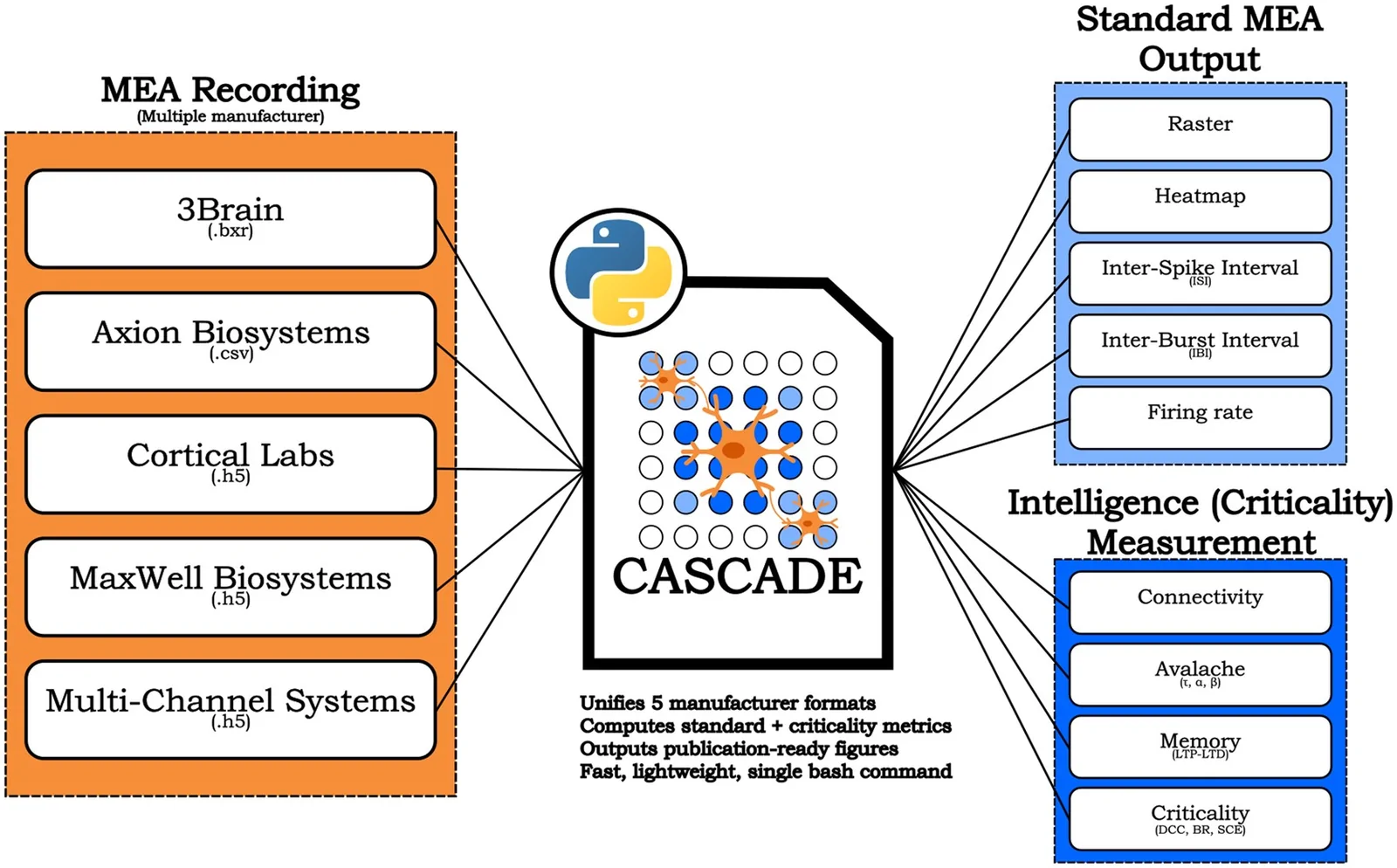

## 1 Introduction

Multi-electrode arrays (MEA) are devices that contain multiple electrodes that can receive, measure, and record electrical signals (input) as well as send and stimulate electrical signals (output) (Thomas *et al.* 1972, Gross 1979). In biological applications, MEAs are used to observe and stimulate excitable cells or tissues such as neurons (Williams *et al.* 2025), cardiac cells (Kussauer *et al.* 2024), muscle cells (Rabieh *et al.* 2016), and other excitable cells. In neuroscience, MEAs record extracellular action potentials simultaneously across dozens to thousands of electrodes, making them among the most widely used platforms for characterizing network-level activity in cultured neurons (Williams *et al.* 2025), brain slices (Mapelli *et al.* 2025), and brain organoids (Huang *et al.* 2022).

Since the invention of the MEA, a variety of MEAs have been manufactured by different brands: 3Brain, Axion Biosystems, Cortical Labs, MaxWell Biosystems, Multi-Channel Systems (MCS), etc. However, each manufacturer has a different recording file format with a distinct binary format and has their own proprietary software to analyze the recordings (Hu *et al.* 2022). Resulting in an unstandardized and fragmented computational landscape in which reproducibility and cross-platform comparisons are structurally difficult to achieve. In general, propriety MEA analysis software measures: spike, inter-spike-interval (ISI), burst, inter-burst interval (IBI), firing rate frequency, and network. Although those measurements offer essential insights into neuronal activity, further parameters are needed in order to comprehensively understand the MEA recordings.

One of the hallmarks of MEA technology and bioinformatics research is the creation of the “brain dish” by (Kagan *et al.* 2022). The MEA device was programmed so that neuron cells could play the Pong game and measure intelligence at the neuronal level. The mathematical foundation and measurement of an intelligence at the neuronal level is by the neuronal avalanches (Beggs and Plenz 2003). The neuronal avalanches have a critical point, networks exhibit power-law distributed neuronal avalanche size and duration distributions, optimal information transmission, and maximal dynamic range (Beggs and Plenz 2003, Shew and Plenz 2013). Quantitative metrics derived from these distributions, including the size exponent *τ*, the duration exponent *α*, the predicted versus fitted exponent ratio (*β*), the Deviation from Criticality Coefficient (DCC), and the Branching Ratio (BR), together provide a multidimensional fingerprint of how close a network operates to criticality (Wilting and Priesemann 2018, Habibollahi *et al.* 2023). In addition, the shape collapse error (SCE) offers a continuous measure of avalanche universality that extends beyond individual exponent estimates (Friedman *et al.* 2012).

Since existing open-source MEA analysis tools, including MEA-ToolBox (Hu *et al.* 2022), MEAanalysis (Gordon *et al.* 2025), and autoMEA (Hernandes *et al.* 2024), are each restricted to a single manufacturer and do not extend to criticality (intelligence) analysis (Table S1, available as supplementary data at *Bioinformatics* online). In order to address these limitations, we present CASCADE (Criticality Avalanche Spike Cross-platform Analysis Detection Engine), a modular and robust Python-based command-line (bash) pipeline. CASCADE was made to be robust and can process and calculate standard and criticality analysis MEA recordings from multiple MEA manufacturers. CASCADE addresses a gap that none of these tools individually resolves.

**Table 1:** CASCADE pipeline performance across MEA recordings.

MEA	Recording					Performance	
	Size	Duration (s)	Active electrodes	Spikes	Avalanches	Run time (s)	Peak memory (MB)
3Brain^a^	1.1 GB	70	142	9636	421	16.1	1570
Axion	337 KB	181	70	12 286	898	6.4	250
Cortical Lab	589 MB	192	33	3515	520	4.8	249
Cortical Lab^b^	8.6 GB	2702	59	679 908	27 288	12.4	353
MaxWell	1.6 GB	300	341	82 610	9197	20.3	391
MCS	502 MB	315	43	22 591	705	5.2	251

a 3Brain device is a high-definitiotab MEA (HD-MEA), with 4077 active electrodes.

b CL1 recording in Pong game mode (Kagan *et al.* 2022).

## 2 Materials and methods

**Figure 1:**
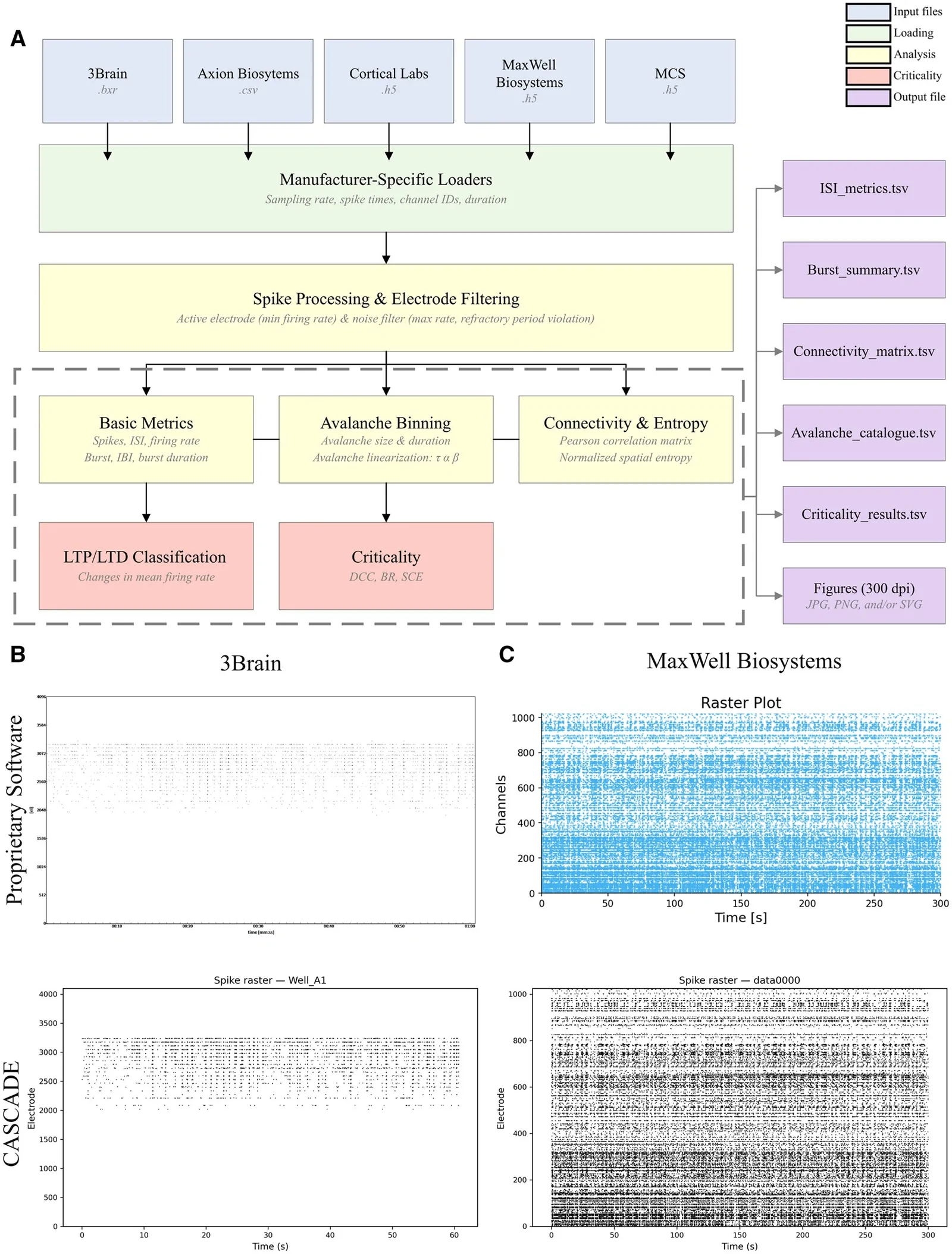
Overview of the CASCADE analysis pipeline and validation against proprietary software. (A) Block-flow diagram of the CASCADE pipeline from raw input files to output TSV files and figures. (B and C) Raster plots from proprietary software (top) and CASCADE (bottom) for 3Brain (B) and MaxWell Biosystems (C) recordings.

CASCADE is implemented in Python (≥3.10) and organized into three layers: loaders, core modules, and utilities. In order to enforce a clean separation between file input and computation, each manufacturer loader converts raw recording files into a single standardized data structure shared across all downstream analysis. Since core modules never access raw files directly, extending the pipeline to a new manufacturer requires writing only one new loader without modifying any existing analysis code. CASCADE operates on manufacturer-exported spike times rather than raw voltage traces, all subsequent analysis is conducted on post-detection spike data, and voltage-level processing such as filtering or spike detection falls outside the scope of the pipeline. Overall CASCADE coding pipeline are described in block-flow diagram format in Fig. 1A.

Six MEA recordings from five different MEA device companies were used to develop, test, and benchmark CASCADE (available at Zenodo: https://doi.org/10.5281/zenodo.19161615). BioCAM DupleX was the model used for the 3Brain device; Maestro MM was the model used for the Axion Biosystems device; CL1 was the model used for the Cortical Lab device; MaxOne was the model used for the MaxWell Biosystems device; and MEA2100 was the model used for MCS. CASCADE, currently, supports five manufacturers: 3Brain (bxr is a form of h5), Axion Biosystems (spike.csv), Cortical Labs (h5 from normal recording and Pong game recording), MaxWell Biosystems (h5), and MCS (h5). Although each manufacturer stores spike data in a distinct binary format, HDF5 variants for 3Brain, Cortical Labs, Maxwell Biosystems, and MCS; and a spike-list CSV for Axion Biosystems. SD-rat primary cortical neuron cultures were used in Axion Biosystems, Cortical Lab, and MCS MEA recording and human brain organoids were used in 3Brain and MaxWell Biosystems MEA recording. All loaders convert their respective formats into the same unified structure, ensuring that every subsequent analysis step is platform-agnostic. All loaders apply optional time windowing, so analysis can be confined to any specified interval within the recording.

Following file loading, CASCADE applies a two-stage electrode filter to retain only electrodes with genuine neural activity. First, channels below a minimum firing rate threshold are excluded. For strict filtering of active electrodes, a minimum of 5 spikes per minute is recommended and we use this parameter in this manuscript (--ae 5). Since hyperactive electrodes in MEA recordings frequently reflect electrical noise rather than true neural spiking, a second filter optionally removes channels exceeding a maximum firing rate or a maximum refractory period violation (RPV) percentage. RPV is defined as the fraction of inter-spike intervals shorter than 2 ms, a threshold below which physiological action potential generation is impossible. Thus, the filtering stage separates biologically meaningful signals from electrical artifacts before any further computation.

Basic network activity metrics: spike counts, ISI distributions, firing rates, network burst duration, IBI, and burst spike count; are computed from the filtered spike trains. Network bursts are identified using an ISI-threshold algorithm applied to the pooled population spike train, consistent with the MEAanalysis (Gordon *et al.* 2025).

Neuronal avalanche detection follows the method described by (Habibollahi *et al.* 2023), including a population activity percentile threshold of 0.25 and we use this parameter in this manuscript (--perc 0.25) to define active bins. Spikes are binned at a time resolution equal to the mean inter-electrode interval, and avalanches are defined as consecutive non-empty bins bounded by empty bins (Beggs and Plenz 2003). Size exponent *τ* and duration exponent *α* are estimated by truncated power-law maximum likelihood estimation via KS-minimization. The DCC, BR, and SCE are then derived from the fitted distributions, following (Friedman *et al.* 2012, Wilting and Priesemann 2018, Habibollahi *et al.* 2023), respectively. Together, these metrics constitute a multidimensional criticality fingerprint for each recording.

Functional connectivity between electrode pairs was quantified using the Pearson correlation coefficient computed on binned spike-count time series, with pairs exceeding an absolute correlation threshold classified as functionally connected. In order to characterize how broadly spiking activity was distributed across the array over time, spatial entropy was computed in consecutive time windows, where higher entropy reflects uniform spatial recruitment across active electrodes and lower entropy indicates spatially concentrated activity (Shew *et al.* 2011).

In order to classify plasticity dynamics, the criticality metrics are subsequently passed to cascade-ltpltd, an accompanying R module that classifies each recording as exhibiting long-term potentiation (LTP) or long-term depression (LTD) based on the trajectory of criticality parameters across experimental conditions. The full analysis, from raw file to criticality classification, is executed with a single command-line call.

For ease of use, CASCADE can generate multiple figures at 300 dpi resolution with multiple file formats (JPG, PNG, and SVG). In addition, CASCADE generates tab-separated value (TSV) files that contain calculated metrics, which can be used for further custom analysis that are not included in CASCADE or using other methods that CASCADE does not use. With the TSV files, the user can also customize the figure based on the TSV (Fig. 1, available as supplementary data at *Bioinformatics* online).

## 3 Result

CASCADE result was compared and validated by comparing the result with proprietary software. MEA recordings from 3Brain (software: BrainWave5 v.5.7) and MaxWell Biosystems (MaxLab Live Scope 24.2.4) results were compared (Fig. 1B and C).

To evaluate computational scalability, CASCADE was timed across all six recordings spanning the full range of supported manufacturers and file sizes, from 337 KB (Axion Biosystems spike-list CSV) to 8.6 GB (CL1 pong game recording), using identical parameters on a standard laptop (Intel Core i5-7300U, 2.60 GHz, 7.6 GB RAM). The parameters were --ae 5 only electrodes with 5 spikes in a minute pass the filtering stage; --perc 0.25 where a bin is considered active when the population firing rate exceeds the 25th percentile threshold (Habibollahi *et al.* 2023). The overall CASCADE performance, the run time (Wall-clock) and peak memory, and overall analysis result are calculated (Table 1). CASCADE completed the full analysis pipeline from raw file loading through criticality classification and figure generation in 5–20 s across all recordings. The longest runtime (20.3 s) corresponded to the MaxWell Biosystems recording, which contained 341 active channels and 82 610 spikes, the highest spike density of all recordings tested. Peak memory usage ranged from 250 MB (small recordings) to 1570 MB for the 3Brain recording, and the 8.6 GB CL1 game file required only 352 MB of peak memory, reflecting efficient sparse access to HDF5 spike-time data. Since CASCADE operates on manufacturer-exported spike times rather than raw voltage traces, these times represent the full post-detection analysis overhead. Compared to previous open-source software, MEA-ToolBox (MATLAB-based), it took 30 min to process a 10 min MCS recording with 288 electrodes (Hu *et al.* 2022). Since MEA-ToolBox processes raw voltage traces including spike detection, the longer runtime reflects the additional computational overhead of voltage-level processing; whereas CASCADE operates on manufacturer-exported spike times.

## 4 Conclusion

CASCADE demonstrates that a unified, modular Python pipeline can process MEA recordings from five distinct manufacturers using a single command, eliminating the manufacturer-specific scripting that currently fragments MEA analysis workflows. CASCADE outputs have been validated and are directly equivalent to the current methodological standard for MEA criticality analysis. Thus, cross-platform and cross-laboratory comparisons can be conducted without introducing computational discrepancies attributable to software differences.

Several limitations warrant acknowledgement. CASCADE currently supports five manufacturers, and support for additional platforms would require the implementation of additional loaders. Since CASCADE is implemented in Python as a fully scriptable pipeline, it can be executed with a single command, integrated into shell scripts and cluster environments, and extended to new manufacturers by implementing one loader file of approximately 150–190 lines sub classing a 47-line abstract base class, without modifying any existing analysis code. Thus, the architectural design of CASCADE directly lowers the cost of cross-platform MEA analysis and provides a reproducible computational pathway that is manufacturer-agnostic from raw file input to criticality classification.

Arguably, the most significant contribution of CASCADE is not any individual analytical method, but the standardization of a reproducible computational pathway from raw manufacturer files to criticality classification. Thus, CASCADE has its own advantages and use compared to manufacturers’ proprietary software and existing open-source software. Reflecting the advancement of DNA sequencing, where data output and analysis software became manufacturer-agnostic over time, CASCADE represents a step toward the same standardization for MEA technology. In order to further advance this goal, future versions will integrate additional manufacturer support, real-time streaming analysis, and expanded connectivity metrics. Furthermore, the cascade-ltpltd R module for LTP/LTD classification will be formally documented and released as part of the CASCADE package, completing the pipeline from raw recording to plasticity classification.

## Supplementary Material

btag570_Supplementary_Data

## Data Availability

CASCADE is freely available at GitHub repository (https://github.com/FA387/cascade-mea) and can be installed via PyPI (pip install cascade-mea). The source code is archived at Zenodo (https://doi.org/10.5281/zenodo.20840795). Data used in this work are available at Zenodo repository (https://doi.org/10.5281/zenodo.19161615). The data underlying this article are available in the article and in its online supplementary material.
