# Supplementary material for: CASCADE: criticality avalanche spike cross-platform analysis detection engine, a multi-manufacturer MEA bash analysis pipeline
Source: Bioinformatics. 2026 Jul 29;42(8):btag570. doi: 10.1093/bioinformatics/btag570 (PMC13478863; doi:10.1093/bioinformatics/btag570)
Supplement: Supplementary file 1 [file btag570_supplementary_data.docx]

APPLICATION NOTES – SUPPLEMENTARY FILE

**CASCADE: Criticality Avalanche Spike Cross-platform Analysis Detection Engine, a Multi-Manufacturer MEA Bash Analysis Pipeline**

Forbes Avila^1,2^, Jin Kim^3,4^, Jong-Chan Park^5, 6, 7^, Rian Kang^7^, Hyunsu Lee^8,9,10,*^, Sun-Hyun Park^1,2,*^

^1^ Center for Alternatives to Animal Testing, Korea Institute of Toxicology, Yuseong, Daejeon 34114, Republic of Korea

^2^ Human and Environmental Toxicology, University of Science and Technology (UST), Yuseong, Daejeon 34113, Republic of Korea

^3^ Department of Physiology, College of Medicine, Soonchunhyang University, Cheonan, 31151, Republic of Korea

^4^ Institute for Molecular Metabolism Innovation, Soonchunhyang University, Asan, 31538, Republic of Korea

^5^ Department of Biophysics, Institute of Quantum Biophysics, Sungkyunkwan University, Suwon 16419, Republic of Korea

^6^ Department of MetaBioHealth, Sungkyunkwan University, Suwon 16419, Republic of Korea

^7^ Department of Biopharmaceutical Convergence, Sungkyunkwan University, Suwon 16419, Republic of Korea

^8^ Department of Physiology, School of Medicine, Pusan National University, Yangsan-si 50612, Republic of Korea

^9^ Research Institute for Convergence of Biomedical Science and Technology, Pusan National University Yangsan Hospital, Yangsan-si 50612, Republic of Korea

^10^ Medical Research Institute, School of Medicine, Pusan National University, Yangsan-si 50612, Republic of Korea

* To whom the correspondence should be addressed

**Table S1.** Comparison of CASCADE and Existing Open-Source MEA Analysis Tools.

| **Feature** | **CASCADE** | **MEA-ToolBox** | **MEAanalysis** | **autoMEA** |
| --- | --- | --- | --- | --- |
| Language | Python | MATLAB | R | Python |
| Interface | Bash | GUI | R console | GUI |
| MEA Manufacturer | 3Brain, Axion, Cortical Lab, MaxWell, MCS | MCS | Axion | MCS |
| Input type | Spike times | Raw voltage | AxIS CSV | Raw voltage |
| Standard network metrics | ✓ | ✓ | ✓ | ✓ |
| Criticality/avalanche analysis | ✓ | ✗ | ✗ | ✗ |
| Open source | ✓ | ✓ | ✓ | ✓ |
| Link | https://github.com/FA387/cascade-mea | https://github.com/DrJPFrimat/MEA-ToolBox | https://github.com/egordon2/MEA-analysis-package | https://gitlab.com/QMAI/papers/autoMEA |

**Supplementary Fig. 1.** Data and Figures Generated from CL1 Game MEA Recording using CASCADE.
A) Avalanche size plot. B) Avalanche duration plot. C) Linearized avalanche size plot. D) Linearized avalanche duration plot. E) Active area plot. F) Population firing rate. G) Spatial entropy plot. H) Network burst. I) Electrode connectivity plot. J) Raster plot. K) Rate heatmap. L) LTP-LTD comparison.
